# Numerical and experimental study of pyrophoric activated metal Mg surface combustion characteristics

**DOI:** 10.1098/rsos.172064

**Published:** 2018-05-16

**Authors:** Hesong Huang, Zhongxiang Tong, Chaozhe Wang, Biao Wang

**Affiliations:** Aeronautics and Astronautics Engineering College, Air Force Engineering University, Xi'an, 710038, People's Republic of China

**Keywords:** pyrophoric, activated metal, diffuse, heat balance equation, CFD

## Abstract

The combustion of multi-hole pyrophoric activated metal is solid combustion and the combustion mechanism is quite complex, which is a difficult problem to be solved. Once the pyrophoric activated metal is exposed to air, the oxygen diffuses to the interior of the activated metal within plenty of holes and reacts with it, which enlarges the contact area with oxygen. Consequently, the whole combustion is vigorous and the temperature rises rapidly. To study the combustion mechanism of the chaff, the surface heat balance equation is established in this work by taking Mg as the activated metal. To solve this equation, the chaff adiabatic wall temperature distribution is computed by computational fluid dynamics in the presence of high-speed airflow. Then, the chaff surface temperature distribution is obtained by solving the heat balance equations. Finally, numerical and experimental results obtained via an infrared thermal imager are compared to demonstrate the effectiveness of the established equation.

## 1. Introduction

A surface-type infrared (IR) decoy is compressed by thousands of strips of chaff. The chaff is composed of multi-hole activated metal. These diffuse rapidly in the presence of high-speed airflow and then combust in the air. Hence, the radiation characteristics of the surface-type IR decoy is affected by the combustion of multi-hole activated metal. This results in an active research field for the combustion of multi-hole activated metal.

There exist plenty of holes in the multi-hole activated metal, which increases to a significant degree the surface area of activated metal. Oxygen diffuses to the interior of the chaff through the holes in the multi-hole activated metal; then the activated metal is exposed to air, which enlarges the contact area and increases the pyrophoric rate of the chaff. In this way, the radiation intensity is quite strong in the pyrophoric process of activated metal.

Different surfaces of the chaff are influenced by aerodynamic heating in the high-speed motion, which results in the surface temperature distribution of each surface of the chaff varying during the pyrophoric process of activated metal chaff. The oxygen diffuses to the interior of the activated metal via plenty of holes and reacts with it. Therefore, the combustion rate of activated metal is mainly related with the oxygen diffusion rate. The oxygen diffusion rate is related with the diameter of the holes, the ratio of the volume occupied by the holes to the volume of the activated metal material, the surface area of the chaff and the transportation rate of oxygen to the activated metal. The activated metal determines the value of reaction enthalpy and how much energy is generated by combustion, which have an impact on the combustion process and temperature ultimately. The velocity of airflow impacts the concentration of oxygen on the surface of the chaff, and impacts on the reaction rate consequently. The pyrophoric reaction of the activated metal is not only related with chaff velocity but also with the activated metal material [1].

The combustion of multi-hole activated metal is solid combustion and the combustion mechanism is quite complex; there are many studies on it. Wang *et al*. [2] ignited Al and Ti powder particles by laser beam. Al and Ti particles were carried by the airflow. The airflow pattern varies from laminar to turbulent flow. The flow pattern was driven by a computational fluid dynamics (CFD) model. The brightness of the streak and the combustion temperature of Al were substantially decreased in turbulent flow, while the reduction was minor for Ti compared with Al. The combustion rate increased for both Al and Ti in turbulent flow. Wiharm [1] established the combustion model of activated metal Fe. The maximal combustion temperature could be obtained by the model. The combustion temperature increased with velocity and reduced with altitude. Zenin *et al*. [3] put Al–Mg alloy particles into a free-falling combustion chamber and ignited it with a ruby laser. They studied the impact of different ratios of Al and Mg, different pressures and different media on the combustion. The combustion time, luminescence pulsations, fragmentation and combustion products were tested. Corcoran *et al*. [4] ignited the Al and Mg powder in water vapour. The effect of oxygen and other oxidizing species could be neglected. They found that the larger size particles burned longer. Gao *et al*. [5] studied the primary and secondary combustion of Al–Mg alloy. The products of primary combustion were collected to simulate the secondary combustion in the high-temperature water vapour tube furnace. They found that the Mg in the Al–Mg alloy combusted first and the Al combusted later. Koch *et al*. [6] studied the combustion of Yb in oxygen. Yb would yield intense luminous flames via adding the consolidated stoichiometric mixtures in the combustion. It is claimed that the flame temperature of Yb in oxygen was in the same range as in Mg-based systems. Yang *et al*. [7] proposed a new method for continuously synthesizing metal oxide nanoparticles by injecting micro-sized metal powder precursors into the flame. They found that the sizes of oxide nanoparticles were correlated with the flame temperatures and residence times. Chen *et al*. [8] designed a new IR/ultraviolet dual colour decoy, which contained oxidant, fuel, energetic binder and additives. The new dual colour decoy changes the spectral radiation characteristics of the traditional IR decoy.

Most previous studies are about the combustion characteristics of activated metal; however, less attention is paid to pyrophoric activated metal. The combustion model is established in [1], while the reduction of activated metal material with time is not considered in the combustion. We can only obtain the maximal temperature by the model. In this paper, the surface combustion model of the activated metal is established based on the work in [1]. Two new factors are taken into account, namely the variation of the mass of the activated metal with oxygen consumption and the impact of the adiabatic wall computed by CFD on the combustion temperature. Then, the combustion temperature of each surface at arbitrary time and the temperature gaps between different surfaces can be obtained via the proposed model.

Mg, as a classical kind of pyrophoric activated metal, is used to study activated metal chaff surface combustion characteristics in this work. The surface heat balance equation of the activated metal is established. Then, the accuracy of the model is verified by experiment. Furthermore, surface combustion characteristics of pyrophoric activated metal chaff are obtained by numerical computation.

## 2. Surface heat balance equation

**Figure 1.**
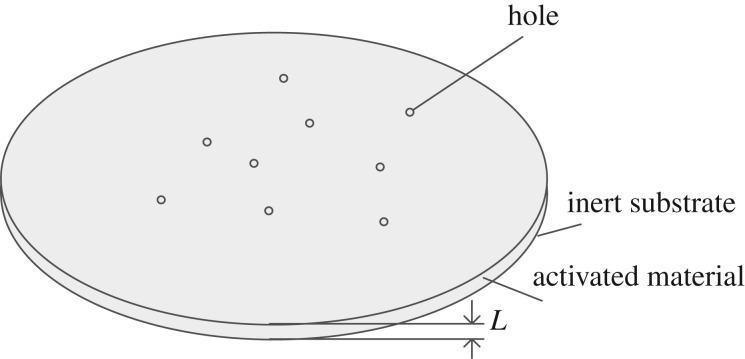
Sketch map of activated metal chaff.

Assuming that the inert substrate material is circular chaff of diameter *D_f_*, the activated metal Mg of thickness *L* uniformly covers the surface of the inert substrate material. There exist plenty of holes within the activated metal, which increases the contact area with oxygen and improves pyrophoric reaction widely. The holes occur in the activated metal with a uniform distribution, which is shown in figure 1. The total number of holes is *n*.

Owing to aerodynamic heating, the temperature and the concentration of oxygen on the surface of the chaff are different. The temperature of the leading edge of the chaff is high and other parts are heterogeneous, which makes the luminance distribution of the chaff different in combustion. The activated metal on the surface of the chaff is divided into *N* surfaces. The heat balance equation of the surface is given in [9]:

2.1 $$m_{i}(t)C_{p}\frac{\text{d}T_{i}}{\text{d}t}=Q_{\mathrm{in}}+Q_{\mathrm{env}}+Q_{\mathrm{con}}-Q_{\mathrm{cv}}-Q_{\mathrm{rad}},$$

where *Q*_in_ is the energy generated by the activated metal, *Q*_con_ is the energy generated by the heat conduction among surfaces of the chaff, *Q*_env_ is the energy generated by environment radiation, *Q*_cv_ is the energy generated by the heat convection between surface *i* and the air, *Q*_rad_ is the energy generated by the radiation from surface *i*, *T_i_* is the temperature of surface *i* and *m_i_*(*t*) is the mass of surface *i* at time *t*, which is given as follows:

2.2 $$m_{i}(t)=m\frac{L_{i}(t)A_{i}}{LA},$$

where *m* is the initial mass of the activated metal, *A_i_* is the area of surface *i*, *A* is the total area of the chaff, which is $A=\pi D_{f}^{2}/4$, and *L_i_*(*t*) is the thickness of surface *i* at time *t*, which is given as

2.3 $$L_{i}(t)=L-\frac{ALkW_{i}(t)M_{\mathrm{Mg}}}{mA_{i}},$$

where *W_i_*(*t*) is the total oxygen consumption of surface *i* at time *t*, *M*_Mg_ is the mole mass of Mg and *k* is the consumption ratio between the activated metal and oxygen.

Heat conduction *Q*_con_ is mainly generated by the temperature gap between the surface and other surfaces nearby. The adiabatic wall temperature gap is small between the surface and other surfaces nearby; thus *Q*_con_ is quite small, which could be neglected [10,11].

To solve equation (2.1), the combustion energy *Q*_in_ is deduced first.

Because of the energy generated by consuming activated metal, there exists the balance of mass and energy inside the multi-hole activated metal. The balance correlation relies on the concentration of oxygen and the temperature of the activated metal. The balance relationship of oxygen should be considered, which satisfies the following equation [1]:

2.4 $$\omega \frac{\text{d}C}{\text{d}t}=D\frac{\partial^{2}C}{\partial z^{2}}-\frac{n\pi D_{h}}{A}k_{s}C(z),$$

where *z* is the distance between an arbitrary point in the activated metal chaff and the inert substrate, and *z* = *L*(*t*) is the surface of the activated metal; *z* = 0 is the surface of the inert substrate material; *C*(*z*) is the oxygen concentration of the holes within the activated metal, and the distance of the inert substrate is *z*; *k_s_* is the reaction rate; *D* is the diffusion coefficient of oxygen, which is $D=D_{h}\sqrt{RT}/6\sqrt{\pi }$_;_ and *ω* is the ratio of the volume occupied by the holes to the volume of the activated metal material, which satisfies the following equation:

2.5 $$\omega =\frac{nD_{h}^{2}}{D_{f}^{2}},$$

where *D*_h_ is the diameter of the hole.

Assuming that the concentration of oxygen reaches invariance quickly in the combustion, which is

2.6 $$\frac{\text{d}C}{\text{d}t}=0.$$

It can be deduced from equation (2.4) that

2.7 $$\frac{{\text{d}}^{2}C}{\text{d}z^{2}}=\frac{n\pi D_{h}k_{s}C(z)}{AD}.$$

The concentration of oxygen and the reaction rate are the lowest on the surface of the inert substrate material, which is *z* = 0. As the oxygen cannot diffuse to the surface of the inert substrate material, d*C*/d*z*|*_z_*_=0_ = 0. However, *z* = *L*(*t*) is the surface of the activated metal, and the concentration of oxygen and the reaction rate are the largest at *z* = *L*(*t*). There are two forms for the mass flux of oxygen transported to the surface of the activated metal: one is bulk diffusion and the other is bulk mass transport. These forms satisfy the following equation:

2.8 $$D\frac{\text{d}C}{\text{d}z}{|}_{z=L}=k_{m}(C_{{\text{O}}_{2}}-C(z)),$$

where *k_m_* is the mass transfer coefficient, which is given as follows:

2.9 $$\left\{ \begin{array}{l} k_{x}=0.182\left(\frac{p_{0}}{p}\right){\left(\frac{T}{T_{0}}\right)}^{1.81}\\ k_{m}=0.664\sqrt{\frac{V_{f}}{D_{f}}}\sqrt[{6}]{\frac{\rho k_{x}^{4}}{\mu }} \end{array}\right.,$$

where *ρ* is the density of the atmosphere, *μ* is the kinematic viscosity of air, and *p*_0_ and *T*_0_ are the standard pressure and temperature of the atmosphere, respectively. Double integrating both sides of equation (2.7), we have

2.10 $$C(z)=\frac{C_{{\text{O}}_{2}}\text{ch}(\theta z)}{\text{ch}(L(t)\theta )+\frac{D\theta }{k_{m}}\text{sh}(L(t)\theta )},$$

where *θ* is given as

2.11 $$\theta =\sqrt{\frac{6n\pi k_{s}}{A}}{\left(\frac{RT}{\pi }\right)}^{-1/4}.$$

The primary reaction is the oxidation of Mg in the pyrophoric reaction.

2.12 $$2\text{Mg}+{\text{O}}_{2}\overset{}{⟶}2\text{MgO}+\text{energy.}$$

This reaction generates 1202.9 kJ energy by consuming per mole of molecular oxygen. *Q*_in_ generated by surface *i* is given as follows:

2.13 $$Q_{\mathrm{in}}=\Delta HJ_{{\text{O}}_{2}}S_{i},$$

where Δ*H* is the reaction enthalpy, $J_{{\text{O}}_{2}}$the mass flux of oxygen in unit area, which is $J_{{\text{O}}_{2}}=-D\text{d}C\text{/d}z$; the negative sign indicates that oxygen diffuses to the direction of decreasing concentration. *S_i_* is the area of surface *i*, and *S_i_* = *nπL*(*t*)*D*_h_*A_i_*/*A*.

Oxygen in the atmosphere diffuses to the interior of the activated metal through the multi-hole structure and then reacts with it. This diffusion includes bulk diffusion and bulk mass transport. The mean diameter of the holes is only 1.5 × 10^−8^ m, which is less than the mean free path length of oxygen (the mean free path length of oxygen is 6.59 × 10^−8^ m at standard conditions). The oxygen molecules always collide with the wall of the holes during the entire diffusion, which makes the coefficient of the bulk diffusion quite small. Thus, it is difficult for oxygen molecules to diffuse in the holes of the activated metal. On the other hand, the concentration of oxygen reduces continuously in the process of diffusion, which further decreases the reaction rate.

The integration of equation (2.7) yields

2.14 $$J_{{\text{O}}_{2}}=\int_{0}^{L(t)}\frac{\pi nD_{h}k_{s}C(z)}{A}\;\text{d}z.$$

The energy generated by the combustion of surface *i* is given as

2.15 $$Q_{\mathrm{in}}=\frac{\pi nL(t)D_{h}A_{i}}{A}\int_{0}^{L(t)}\frac{\pi \Delta HD_{h}k_{s}nC(z)}{A}\;\text{d}z$$

and

2.16 $$\frac{\partial W_{i}}{\partial t}=\frac{\pi^{2}n^{2}L(t)D_{h}^{2}A_{i}k_{s}}{A^{2}}\int_{0}^{L(t)}C(z)\;\text{d}z.$$

The energy *Q*_env_ generated by surface *i* is

2.17 $$Q_{\mathrm{env}}=\alpha_{i}\sigma T_{a}^{4}A_{i},$$

where *α_i_* is the absorptivity of surface *i*, *T_a_* is the temperature of the environment and *σ* is the Boltzmann constant, which is 5.67 × 10^−8^ W/(m^–2 ^K^−4^) [12].

The energy *Q*_rad_ radiated to the environment of surface *i* is

2.18 $$Q_{\mathrm{rad}}=\varepsilon_{i}\sigma T_{i}^{4}A_{i},$$

where *ϵ_i_* is the emissivity of surface *i*. It can be known from the radiation law of a black body that *α_i_* = *ϵ_i_*. Assume that the emissivity of all the surfaces is the same in this work.

To discuss the heat convection between surface *i* and the air, not only the heat dissipation effect of high-speed airflow, but also the aerodynamic heating must be considered. Equation (2.19) is the heat convection equation of surface *i*, which contains the aerodynamic heating

2.19 $$Q_{\mathrm{cv}}=A_{i}h_{x}(T_{i}-T_{i}^{w}),$$

where *h_x_* is the heat convection coefficient given as follows:

2.20 $$h_{x}=0.332\sqrt{\frac{\rho V_{f}}{D_{f}}}\sqrt[{6}]{\frac{c_{p}^{2}\lambda_{a}^{4}}{\mu }}.$$

$T_{i}^{w}$ is the adiabatic wall temperature of surface *i*, which is obtained by CFD. The solution of $T_{i}^{w}$ is dealt with in §3 in detail.

The heat balance equation of surface *i* is shown as follows:

2.21 $$\begin{array}{rl} & \frac{C_{p}m_{i}L(t)}{L}\frac{\partial T_{i}}{\partial t}=\frac{\pi^{2}n^{2}L(t)D_{h}^{2}A_{i}k_{s}\Delta H}{A^{2}}\\ & \;\int_{0}^{L(t)}C(z)\;\text{d}z-A_{i}h_{x}(T_{i}-T_{i}^{w})-A_{i}\sigma \varepsilon ({T_{i}}^{4}-T_{a}^{4}). \end{array}$$

Substituting equation (2.10) into the integral of equation (2.21) yields

2.22 $$\Delta T_{i}=\frac{A_{i}L}{C_{p}m_{i}L(t)}\left[\frac{\pi n\Delta HDD_{h}L(t)\theta \tanh(\theta L(t))C(L(t))}{A}-h_{x}(T_{i}-T_{i}^{w})-\sigma \varepsilon (T_{i}^{4}-T_{a}^{4})\right]\Delta t$$

To guarantee numerical precision, the value of Δ*t* should be less than 0.01 s. Then the variation of activated metal temperature with time is obtained. Assuming that the surfaces of the activated metal can be divided into *N* in the combustion, the radiation intensity of surface *i* is given as follows [13]:

2.23 $$I_{\lambda_{1}-\lambda_{2}}=\sum_{i=1}^{i=N}\int_{\lambda_{1}}^{\lambda_{2}}\frac{\varepsilon A_{i}c_{1}}{\pi \lambda^{5}({\text{e}}^{c_{2}/(\lambda T_{i})}-1)}\;\text{d}\lambda .$$

## 3. The computation of chaff adiabatic wall temperature by computational fluid dynamics

Navier–Stokes equations are chosen as control equations and the pressure-implicit with splitting of operators semi-implicit method for pressure-linked equations (PIM-PLE) algorithms are applied to solve the Navier–Stokes equations. Spatial discretization is accomplished through the second-order accuracy linear interpolation method and the finite volume method. Time discretization is done based on the second-order accuracy backward-difference method. The SST turbulence model is applied as the computational model [14,15].

**Figure 2.**
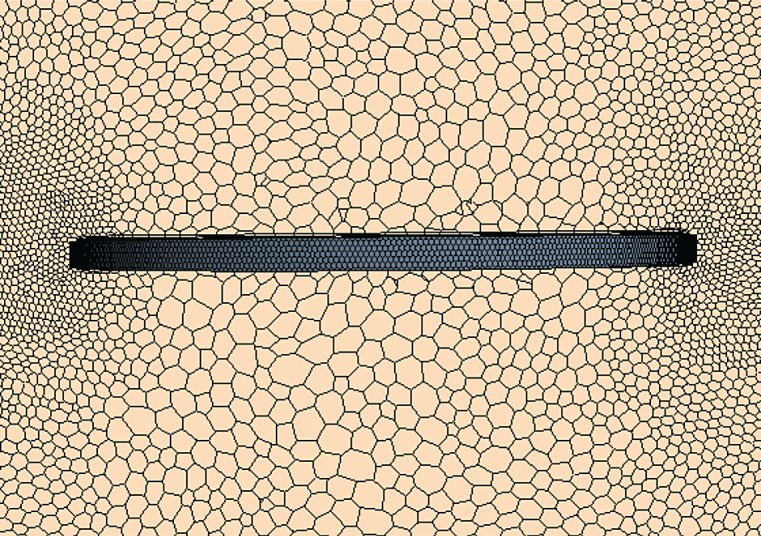
Polyhedral mesh on the surface of the chaff.

Setting the diameter of the chaff as 50 mm, the computational zone is a cube with a side length of 5000 mm. The chaff centre coincides with the centre of the computational zone. The thickness of the first boundary layer is 10^−6^ m and the number of the boundary layers is 12. Polyhedral meshes are used in the computation and the total number of meshes is 2.13 million. The initial altitude and pressure are 0 km and 101 325 Pa, respectively. The environmental temperature is 288.15 K. The polyhedral mesh of the chaff is shown in figure 2.

The computation results at an angle of attack (AOA) of 30° by CFD are shown as follows.

**Figure 3.**
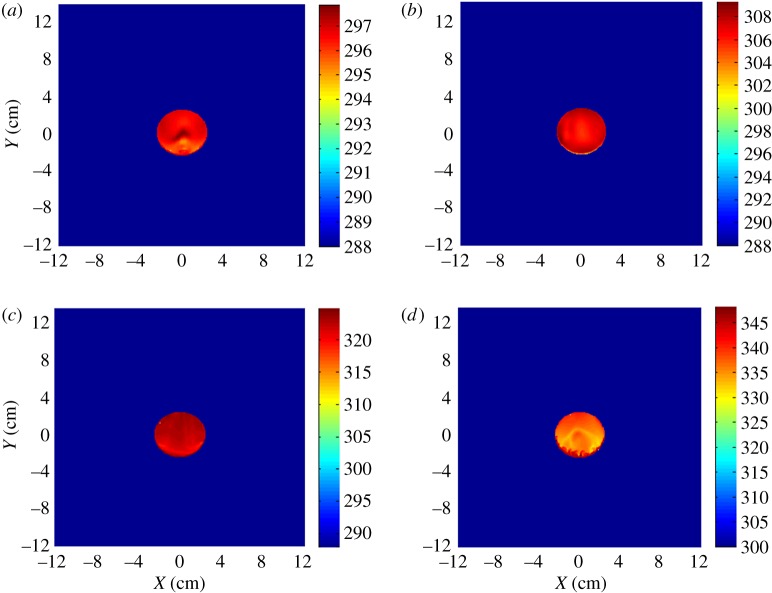
Adiabatic wall temperature destruction of chaff: (*a*) *Ma* = 0.4, (*b*) *Ma* = 0.6, (*c*) *Ma* = 0.8 and (*d*) *Ma* = 1.

It can be seen from figure 3 that the temperature of the leading edge of the chaff is high, and the temperature distribution of other parts is heterogeneous. The flow separation occurred after the leading edge of the chaff at an AOA of 30°, which makes the temperature distribution of the chaff become asymmetrical. The temperature of the adiabatic wall and aerodynamic heating increase rapidly with velocity, so does the gap of the adiabatic wall temperature among surfaces. The gap between the maximal and minimal temperature of the surface reaches 20° with the Mach number *Ma* = 1.

## 4. Comparison of numerical and experimental results

The surface heat balance equation and the adiabatic wall temperature computed by CFD are applied to compute the combustion of pyrophoric multi-hole activated metal in this section. Then, the numerical and experimental results are compared to verify the accuracy.

### 4.1. The chaff temperature test experiment

**Figure 4.**
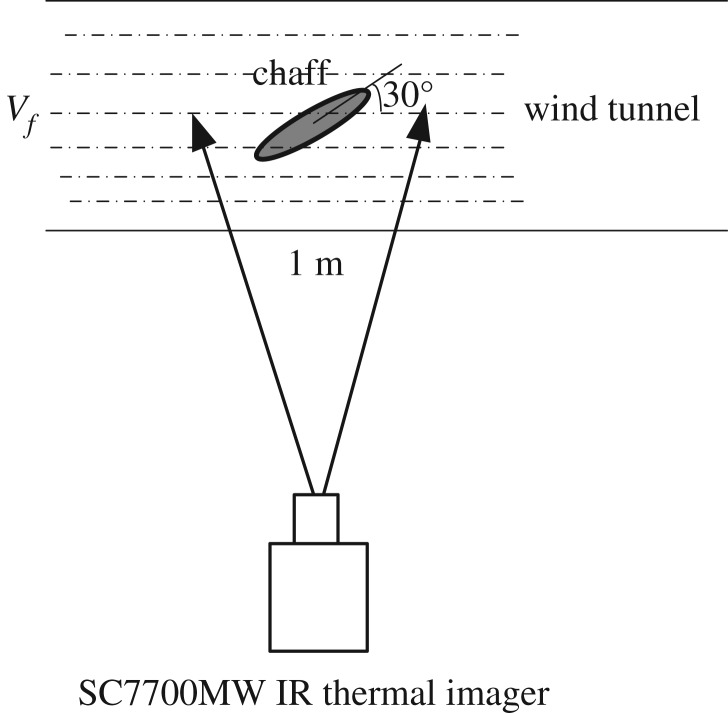
Sketch map of chaff temperature test experiment.

An SC7700MW IR thermal imager is used to test the temperature of the activated metal chaff. The work waveband of SC7700MW is 3–5 µm and field of view is 11° × 8.8°. The activated metal chaff is facing towards the IR thermal imager and the distance between them is 1 m. The diameter of the chaff is 50 mm. The angle between the plane of the chaff and the horizontal plane is 30°. The chaff and IR thermal imager are fixed in a wind tunnel (figure 4).

The National Defense Key Laboratory wind tunnel is used in this work. The wind tunnel length is 2 m. Its cross section is rectangular. The inlet size is 1.2 m × 1.2 m and the outlet size is the same. The flow field quality of the wind tunnel is quite good, and the turbulence intensity is ϵ ≤ 1%. The maximal usable Mach number is *Ma* = 0.6.

The tested IR thermograph is shown as follows.

**Figure 5.**
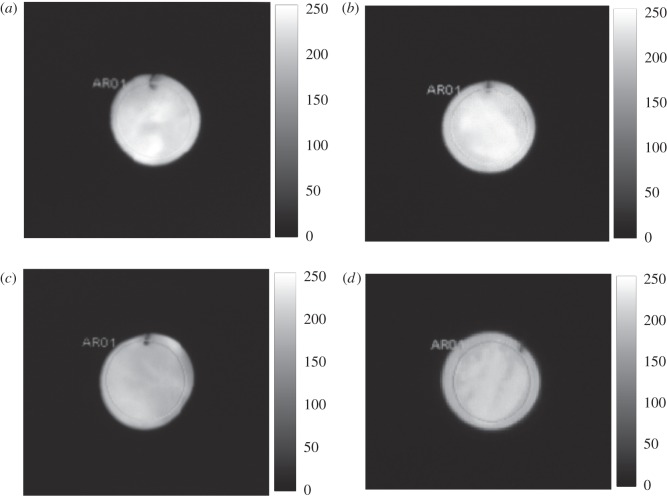
IR thermograph of different velocities at 0.3 s: (*a*) *Ma* = 0.1, (*b*) *Ma* = 0.2, (*c*) *Ma* = 0.4 and (*d*) *Ma* = 0.6.

Figure 5 is the grey level distribution of the activated metal chaff at different velocities obtained by the IR thermal imager. The grey level varies from 0 to 255 [10]. The white area is larger and the corresponding temperature is higher. On the other hand, the black area has opposite characteristics to that in the white area. It can be seen from figure 5 that the white area is the largest with the Mach number *Ma* = 0.2. Therefore, the combustion temperature is the highest. It can be known from §4.3.3 that the activated metal chaff cannot reach the highest temperature at 0.3 s with the Mach number *Ma* < 0.2, where the grey level of the chaff is small. As the Mach number *Ma* > 0.2, the heat convection enhances, while the grey level of the chaff is not as improved as expected.

The activated metal chaff consumes a lot of oxygen in the reaction, and then the concentration of oxygen reduces rapidly. Subsequently, the reaction rate goes down. Once the velocity of airflow increases, the oxygen supply is rapidly increased. Then the concentration of oxygen on the surface of the chaff is increased, and so is the reaction rate. In brief, the pyrophoric reaction rate increases along with the velocity increase.

To the best knowledge of the authors, the combustion temperature of the activated metal chaff is correlated with the energy generated by the activated metal and the heat convection. The reaction rate increases with the velocity increase, while the heat convection increases at the same time. Consequently, the combustion temperature cannot increase with the velocity. This part is analysed in §4.3.

### 4.2. Numerical results

**Figure 6.**
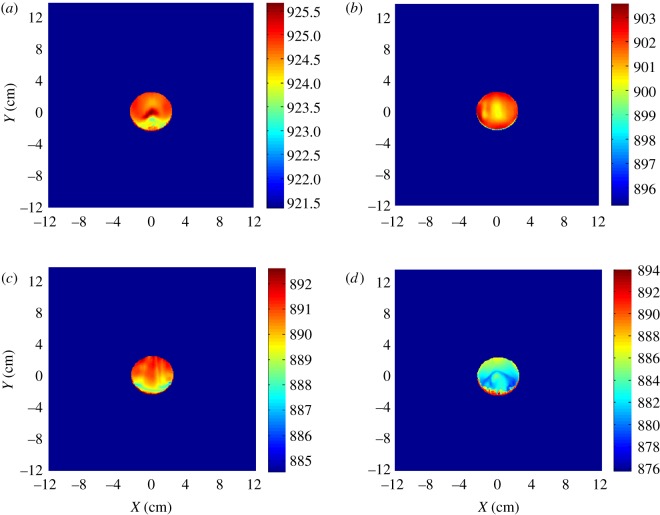
The temperature distribution of chaff at the maximal radiation intensity: (*a*) *Ma* = 0.4, (*b*) *Ma* = 0.6, (*c*) *Ma* = 0.8 and (*d*) *Ma* = 1.

The parameters of the chaff in numerical computation are given in table 1. The temperature distribution of the activated metal chaff is computed by the surface heat balance equation. Figure 6 shows the temperature distribution of the chaff at the maximal radiation intensity in the combustion.

**Table 1.** The parameters of activated metal.

parameter	value	unit
hole diameter, *D*_h_	1.5 × 10^−8^	m
chaff diameter, *D_f_*	0.05	m
rate constant, *k_s_*	0.03	cm s^−1^
emissivity, ϵ	0.8	
activated metal thickness, *L*	5 × 10^−5^	m
hole quantity, *n*	2.34 × 10^12^	
enthalpy of reaction, *ΔH*	601.24	kJ mol^−1^
activated metal mass, *m*	0.6	g

The results (figure 6) show that the combustion temperature distribution is similar to the adiabatic wall temperature distribution. Thus, it is known that the temperature distribution of the adiabatic wall impacts the combustion temperature of the chaff, and the aerodynamic heating cannot be neglected.

### 4.3. Comparison of experimental and numerical results and error analysis

#### 4.3.1. Comparison of the maximal and minimal temperature of the surface

Firstly, the variation of the maximal and minimal temperature of the surface with velocity is computed at the maximal radiation intensity of the chaff in the combustion.

**Figure 7.**
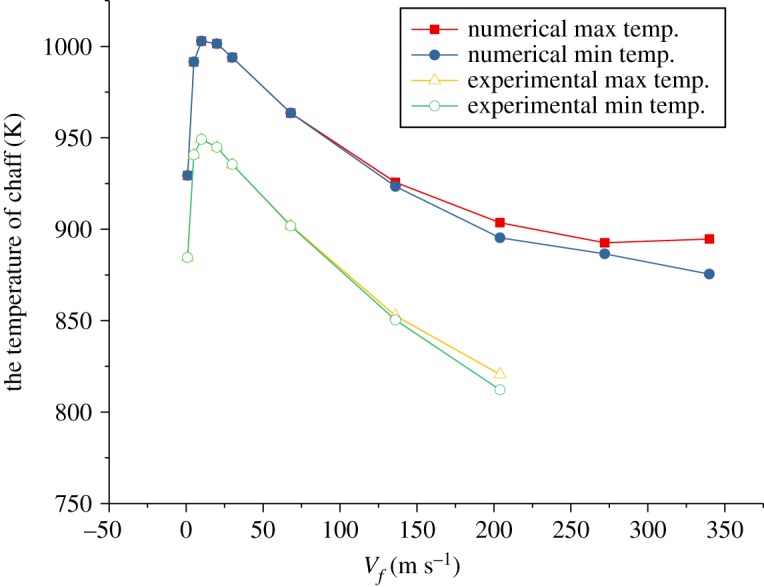
The curve of the maximal and minimal temperatures of the surface at the maximal radiation intensity of the chaff.

The results show (figure 7) that the maximal and minimal temperature increase at first [1], and then decrease with the increase in velocity. The reduction rate becomes smaller and smaller. However, the effect of the aerodynamic heating increases with velocity; so does the heat convection between surfaces and the air. The effect of the aerodynamic heating is not obvious at low velocity and the effect of the heat convection increases with velocity, which makes the temperature of the chaff reduce rapidly. While the effect of the aerodynamic heating becomes obvious with *Ma* > 0.8, the reduction rate of temperature becomes smaller and smaller. The temperature gaps among surfaces increase with velocity, which is in good agreement with the experimental data.

#### 4.3.2. Comparison of radiation intensity of chaff

Then, the total radiation intensity of the chaff is computed, which is compared with the experiment, and the results are shown as follows.

**Figure 8.**
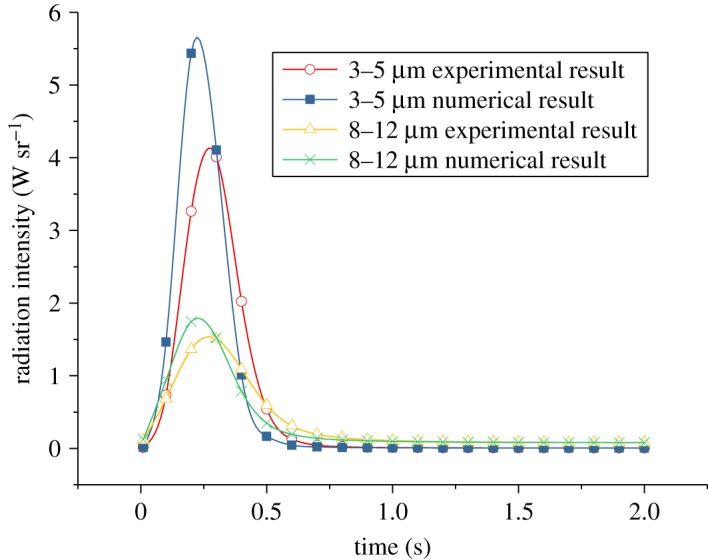
The variation of radiation intensity with the Mach number *Ma* = 0.6.

The comparison of the numerical and experimental total radiation intensity of the chaff (figure 8) shows that the radiation intensity increases to the largest value within 0.25 s, and the combustion is very intense. The radiation intensity in the waveband of 3–5 µm is much larger than 8–12 µm. The numerical results are consistent with the experiment.

#### 4.3.3. The required time for the chaff to reach the maximal radiation intensity

**Figure 9.**
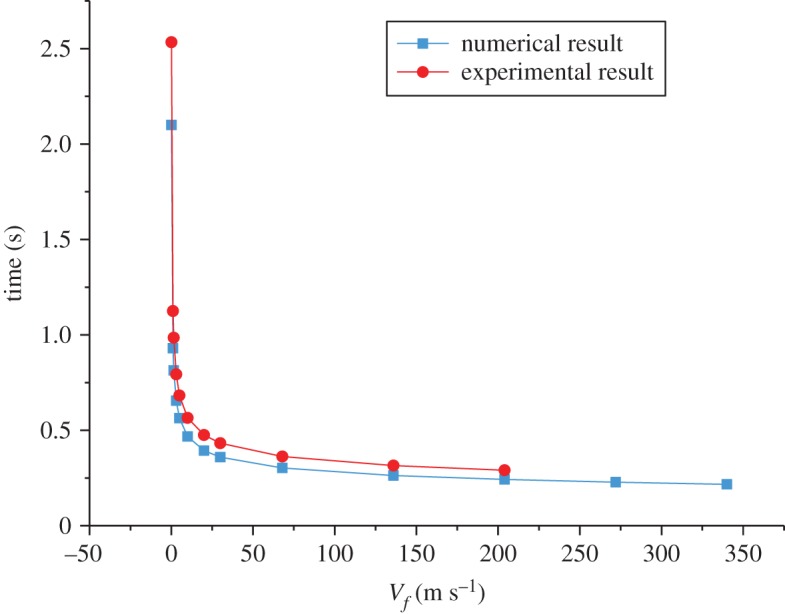
The relationship between time and velocity.

The required time for the chaff to reach the maximum radiation intensity is inversely proportional to the airflow velocity in combustion, and the time is less than 0.5 s when the velocity is faster than 25 m s^−1^ (figure 9). This is consistent with the experiment.

#### 4.3.4. Error analysis

**Figure 10.**
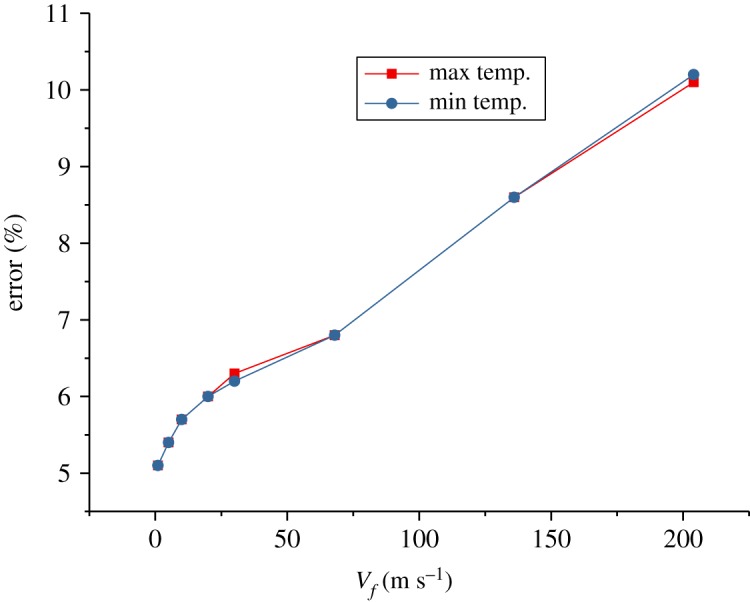
The errors of the maximal and minimal surface temperature.

**Figure 11.**
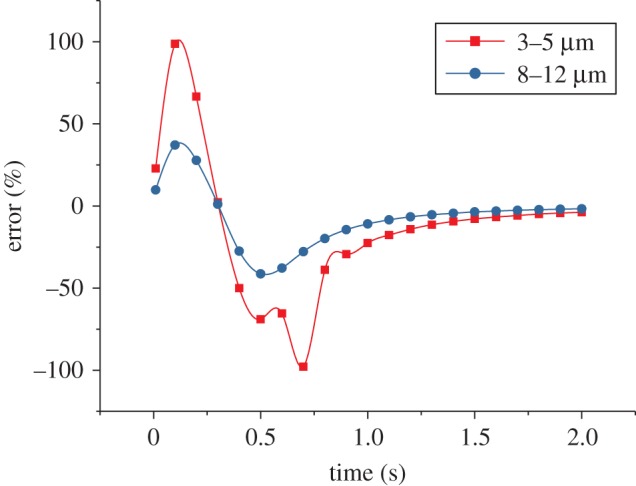
The errors of radiation intensity with the Mach number *Ma* = 0.8.

**Figure 12.**
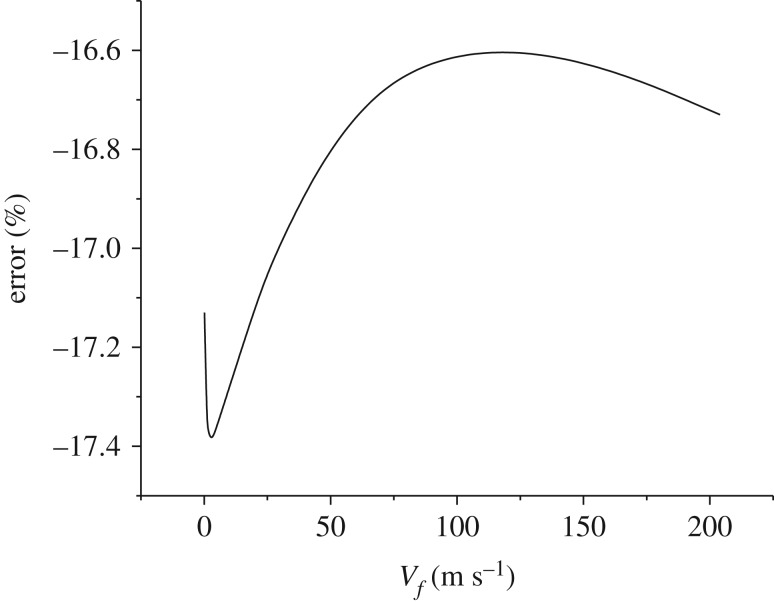
The error of the required time.

The numerical and experimental results in figures 7–9 are compared and the errors are computed. Figure 10 shows the errors of the maximal and minimal surface temperatures at the maximal radiation intensity of the chaff. Figure 11 indicates the errors of radiation intensity with the Mach number *Ma* = 0.8. Figure 12 shows the error of the required time for the chaff to reach the maximal radiation intensity.

As shown in figure 10, the errors of the maximal and minimal surface temperatures are positive, which demonstrates that the combustion rate by numerical computation is higher than that by the experiment, namely the numerical combustion is more vigorous and the temperature is higher compared to the experiment. However, the total amount of activated metal is invariant, with the result that the required time for the chaff to reach the maximal temperature via numerical computation is less than that of the experiment. This is validated in figure 12.

The errors in figure 11 are quite large; the errors rise to 100% initially, then reduce to −100% rapidly and then tend to be stable finally. The errors in figure 11 are the comparison of numerical and experimental radiation intensities at the same time. Compared with the experiment results, the combustion rate of numerical computation is faster, thus the chaff reaches the maximal temperature more quickly. The numerical temperature is always larger than that of the experiment in the increasing phase, so the errors increase rapidly at first. When the chaff reaches the maximal temperature, the required time of the experiment is longer than the numerical one; then the experimental combustion rate is slower than that of numerical computation. Therefore, the surplus activated metal in experiment is more than that from numerical computation, which makes the temperature reduction rate in experiment to be slower. The experimental temperature in the reduction process becomes larger quickly and then the errors decrease. As the combustion is asynchronous, it is inappropriate to compare the numerical radiation intensity with that from the experiment at the same time. Thus, we cannot verify whether the numerical computation results via the comparison of radiation intensity at the same time. Nevertheless, it can be obtained from figures 10 and 12 that the numerical results are quite accurate, and the combustion model is credible.

The reason of the generated errors can be explained as follows:

(1) Dealing with the surface heat balance equation, we assume that the cylindrical holes are vertical with respect to the surface of the activated metal in the numerical computation, which is in favour of the diffusion of oxygen. However, the holes might not be vertical to the surface. Furthermore, there might exist curved holes, which obstruct the diffusion of oxygen and hinder combustion. Thus the pyrophoric rate is higher in the numerical computation.
(2) It is assumed that the holes are uniformly distributed on the surface of the activated metal in numerical computation. However, the distribution of holes might be asymmetrical and the number of holes is an approximation, which may have some difference from reality.
(3) The shape of the hole is considered as cylindrical in numerical computation. However, the shape of the hole is random in reality, with the result that the energy *Q*_in_ generated by surface *i* has some difference from reality.

The comparison of the numerical and experimental results demonstrates that the general values of the numerical computation are in good agreement with the experimental data, the errors are quite small and the accuracy of the numerical model is good. The errors mentioned above will be addressed in the coming research works.

## 5. Conclusion

The surface heat balance equation is established in this work, and the adiabatic wall temperature distribution of the chaff is computed by CFD in the presence of high-speed airflow. Then, the temperature distribution of the chaff is obtained by solving the heat balance equation. Finally, the experimental and numerical results are compared. The comparison shows that the general tendency of the numerical computation is consistent with that of the experiment. This research achieves some brief conclusions as follows:

(1) The maximal temperature of the chaff decreases with the velocity increase and the reduction rate decreases in the presence of high-speed airflow.
(2) The combustion rate increases with the velocity increase, and the required time for the chaff to reach the maximal radiation intensity is less than 0.5 s when the velocity is faster than 25 m s^−1^.
(3) The maximal temperature of the chaff increases initially, and then reduces at the low velocity. The temperature of the chaff reaches the maximum at the velocity 30 m s^−1^.
(4) The temperature distribution of the chaff is asymmetrical; the gaps among surfaces increase with the velocity increase. The gaps among surfaces reach 20° with the Mach number *Ma* = 1.

## Data accessibility

The data in this work are available at Dryad: http://dx.doi.org/10.5061/dryad.1c3m9 [16].
